# Complex systems dynamics in aging: new evidence, continuing questions

**DOI:** 10.1007/s10522-015-9584-x

**Published:** 2015-05-20

**Authors:** Alan A. Cohen

**Affiliations:** PRIMUS Research Group, Department of Family Medicine, University of Sherbrooke, CHUS-Fleurimont, 3001 12e Avenue N, Sherbrooke, QC J1H 5N4 Canada

**Keywords:** Systems biology, Aging, Statistical distance, Physiological dysregulation, Principal components analysis, Emergent property

## Abstract

There have long been suggestions that aging is tightly linked to the complex dynamics of the physiological systems that maintain homeostasis, and in particular to dysregulation of regulatory networks of molecules. This review synthesizes recent work that is starting to provide evidence for the importance of such complex systems dynamics in aging. There is now clear evidence that physiological dysregulation—the gradual breakdown in the capacity of complex regulatory networks to maintain homeostasis—is an emergent property of these regulatory networks, and that it plays an important role in aging. It can be measured simply using small numbers of biomarkers. Additionally, there are indications of the importance during aging of *emergent physiological processes*, functional processes that cannot be easily understood through clear metabolic pathways, but can nonetheless be precisely quantified and studied. The overall role of such complex systems dynamics in aging remains an important open question, and to understand it future studies will need to distinguish and integrate related aspects of aging research, including multi-factorial theories of aging, systems biology, bioinformatics, network approaches, robustness, and loss of complexity.

## Introduction

Twenty-five years ago, Medvedev (1990) outlined more than 300 mechanistic theories of aging. The question then, and the question now, was what to make of this diversity of theories. Is one theory right to the exclusion of all others? Do many mechanisms operate simultaneously? Are some mechanisms downstream and others upstream, such that we might identify one or a few key upstream mechanisms? Do the mechanisms interact with each other, and if so, how?

To some extent, we have answers to some of these questions. For example, very few researchers would now contend that there is a single aging mechanism, though some still argue principally for one central mechanism (Barja 2014). There is both theoretical and empirical evidence for interactions among mechanisms (Kowald and Kirkwood 1996; Ludlow et al. 2014). However, we are still far from a general consensus on a big-picture theory for how mechanisms interact to cause aging (Kirkwood 2011).

One integrative theory proposes a breakdown in interactions within the complex regulatory networks that maintain homeostasis (Ferrucci 2005; Fried et al. 2005). This idea has been around in various forms for a long time, and has many names: homeostenosis (Taffett 2003), allostatic load (Karlamangla et al. 2002; McEwen 1998), and physiological dysregulation (Seplaki et al. 2005). Loss of complexity during aging is a related idea that has also been developed in detail (Lipsitz 2004; Lipsitz and Goldberger 1992; Manor and Lipsitz 2013). These ideas are attractive, and have garnered a fair amount of support, particularly among clinical aging researchers, though they are less familiar to some researchers focused on the molecular mechanisms of aging. However, the challenge has been to accumulate evidence for the importance of such complex systems dynamics in aging. Precisely because the systems are complex, they can be hard to measure. For example, measurement of allostatic load has been questioned as circular (Singer et al. 2004).

Over the last several years, my lab has been working to find ways to test for the presence and importance of complex systems dynamics in aging. We have been doing so at the organism level, and using a particular model of physiological organization. Our approach to complex systems dynamics is described in substantial detail below; briefly, I define complex systems dynamics as changes in the state of complex regulatory networks of molecules that (a) arise due to the structure of regulatory relationships within the network, such as through feedback loops, that (b) cannot be easily understood via simple maps of network structure (i.e., that represent emergent properties of the system), and that (c) may be sensitive to the precise structure of the network and to perturbations in it. Complex systems dynamics might be implicated in aging via a breakdown in the regulatory dynamics (“dysregulation”), through intricate feedback effects among aging mechanisms, or perhaps through other mechanisms, as I will show.

The objective of this article is to summarize our recent findings in an integrative way, and to relate them to the broader literature on complex systems and aging. I thus start with an overview of different ways that complexity has been discussed in the context of aging biology. I continue with a summary of our model of physiological organization, including the evidence we have generated for two particular types of complex systems dynamics: emergent physiological processes (EPPs) and physiological dysregulation. Lastly, I integrate our findings into the larger literature and summarize outstanding questions.

## Approaches to complexity in aging

West and Bergman (2009) proposed an expanded role for systems biology and complexity in aging, but there are many directions this could take, and a lack of clear terminology sometimes leads to confusion. It is important to distinguish complex system dynamics (our approach, detailed in the next section), multi-factorial theories of aging (Kirkwood 2005), systems biology and bioinformatics of aging more generally (de Magalhães and Toussaint 2004; Kirkwood 2011; Soltow et al. 2010), system-level robustness in aging (Kriete 2013), and loss of complexity in aging (Lipsitz 2004; Lipsitz and Goldberger 1992). Each of these research directions provides a critical piece of the puzzle on complexity in aging, and long-term it will be important to integrate them, and perhaps others.

Multi-factorial theories of aging posit simply that aging has many causes (Weinert and Timiras 2003); most aging researchers today would subscribe to this idea at some level. Aging could be multi-factorial but not involve complex system dynamics. For example, there could be a number of different mechanisms that cause damage accumulation, each proceeding largely independently. Even if there are a few specific feedback effects among the mechanisms (e.g. Kowald and Kirkwood 1996), it is not a foregone conclusion that there would be complex system dynamics. The disposable soma theory and related mechanistic theories based on the accumulation of damage, wear and tear, etc. are multi-factorial but do not necessarily imply complex system dynamics (Kirkwood 2005). In fact, the evolutionary mechanisms referred to in the disposable soma theory imply that complex systems dynamics are not central to aging: if they were, aging would likely evolve based on regulatory constraints in complex networks, rather than based on a large number of small trade-offs of things like energy allocation (Cohen Accepted). The distinction between multi-factorial theories and complex systems theories may thus also be important for inferring how aging evolved (see also Kriete 2013; Wensink et al. 2014).

While aging might be multi-factorial but without complex systems dynamics, the reverse is unlikely to be true. Complex systems explanations imply interactions among sub-networks, and it is nearly certain that this would involve complex feedback loops where problems in one system cause problems in another, etc. (Fried et al. 2009; Govindaraju et al. 2014). Multiple systems would thus be implicated in aging, and dysregulatory processes could be started or accelerated from many parts of the network. Furthermore, even if complex system dynamics are important in aging, they are unlikely to be the sole factor. For example, a trade-off between risk of cancer and regenerative capacity appears to be involved in aging (Park et al. 2004). This is likely largely independent of complex systems dynamics.

The complex systems dynamics approach I take fits solidly within a larger systems biology perspective as outlined by Kitano (2002), in which four key features are studied: structure, dynamics, control, and design. I would add function as a fifth feature: what does the system achieve for the organism? In this view, an integrated approach to these features is necessary, though it will not always be possible to conduct research that covers all features. For example, our approach integrates dynamics and function through an understanding of design principles, but there is little emphasis on control or structure. In contrast, Kirkwood (2011) puts an emphasis on computer modeling of dynamics based on detailed knowledge of structure, but does not accord particular importance to higher-order properties of the system. Kriete et al. (2010), (2011) take a similar approach, though higher-order properties have a larger role.

From a different angle, an increasing number of studies are working to map networks (Csermely and Sőti 2006; Hoffman et al. 2014; Xue et al. 2007) and thus establish structure without particular regard to the other features listed. This is part of a more general tendency to use high-throughput technologies to generate and integrate large amounts of data on aging (e.g. de Magalhães et al. 2009; de Magalhães and Toussaint 2004), though such approaches may not really fall within Kitano’s framework for systems biology, absent a link to dynamics, control etc. For example, weighted correlated gene network analysis can take large volumes of biological data and organize it into sub-networks based on the correlations among the molecules. Each sub-network can then be summarized to generate a signal of activity (Langfelder and Horvath 2008; Zhang and Horvath 2005). Such an analysis is very powerful, and can be used for multiple purposes, including an understanding of dynamics as well as for other objectives that may fall outside systems biology. Obviously, the importance of these studies is not determined by whether they can be called “systems biology.” There is a role for all these approaches, including bioinformatics, network analyses, structural maps, computer simulations of network dynamics, and our statistical integration to understand functional dynamics. Nonetheless, imprecision with terms such as complexity, systems, and network, combined with methodological overlap, can sometimes obscure the fact that these approaches pose distinct and complementary biological questions.

**Fig. 1:**
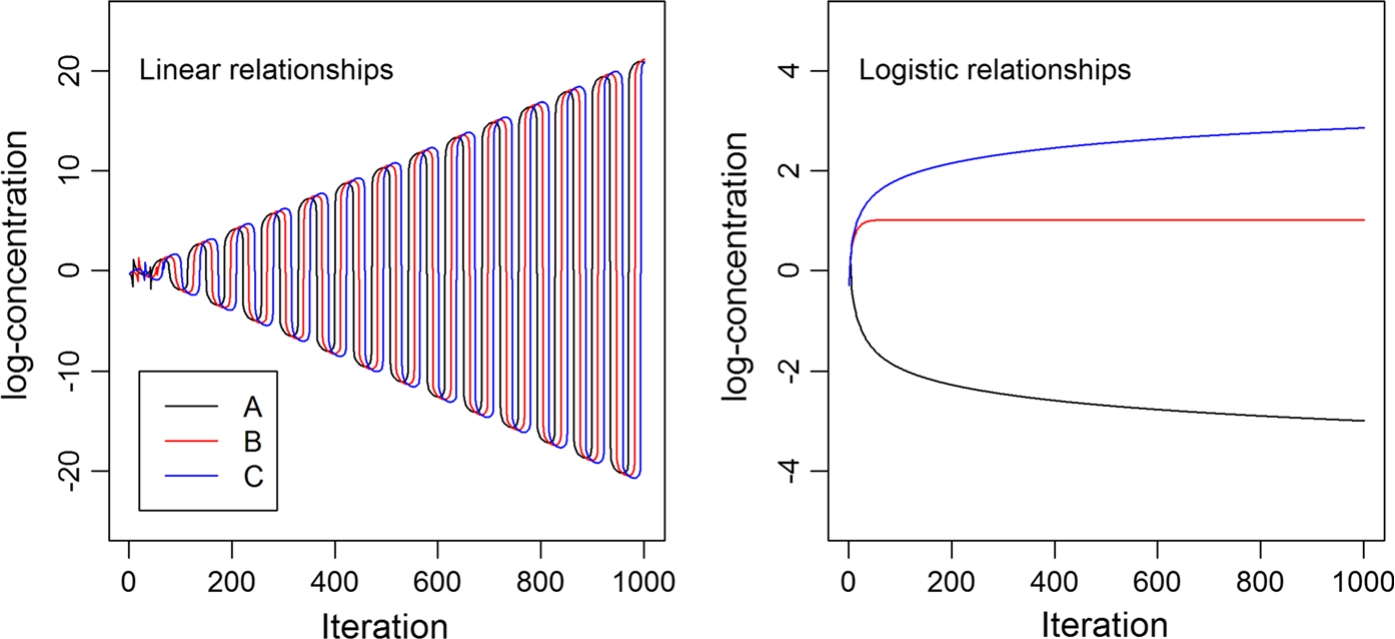
Linear and logistic relationships among molecules can produce vastly different functional dynamics in the system. Results are based on a simple simulation of three molecules, A, B, and C, in which A up-regulates B, B up-regulates C, and C down-regulates A. Over 1000 time steps, linear dynamics produce a highly unstable, fluctuating system, whereas logistic dynamics produce a more stable system. Accordingly, it is not necessarily possible to predict the dynamics of a complex system based solely on a map of what regulates what, without a detailed understanding of the functional forms of the regulatory dynamics

One of the largest challenges with network approaches to aging is the precision needed in order to successfully understand network dynamics (Pearson et al. 2013). In theory, we might build a map of how every molecule relates to every other molecule and then use differential equations to model system behaviour. In practice, we are likely decades away from even identifying all the molecules, much less understanding their dynamic relationships, and it is hard to imagine we will ever have computers powerful enough to run such models. Precisely because the networks are complex dynamic systems, the consequences of missing or slightly erroneous information in network construction are difficult to infer and could be large (Gutenkunst et al. 2007). For example, Fig. 1 shows the very different outcomes in a simple 3-molecule control network based on linear versus logistic functions describing relationships. Fuzzy logic and appropriate sensitivity analyses may partially circumvent such concerns, at least at the cellular level (Kriete et al. 2010). Nonetheless, efforts to understand network function may also require top-down approaches (Pearson et al. 2013), such as those used in our research.

One of the most exciting new directions in complexity in aging research comes from the literature on how robustness is achieved in complex systems (Carlson and Doyle 2000). Robustness is a “property that allows a system to maintain its functions against internal and external perturbations” (Kitano 2007). Following on this literature, Kriete (2013) suggests that in highly optimized systems overall robustness is zero-sum, such that a gain in a certain type of robustness causes a fragility elsewhere. He uses this principle to suggest that evolution of robustness implies the evolution of trade-offs, and thus that aging may be a side effect of other aspects of evolutionary optimization given the specific system-level constraints in complex systems such as organisms. Such an understanding of robustness also integrates well with our understanding of hormetic responses to stress during aging (Rattan 2008).

Lastly, loss of complexity during aging is a specific hypothesis that reflects an understanding of an organism as a complex system (Lipsitz 2004; Lipsitz and Goldberger 1992). Loss of complexity posits that an organism is a sufficiently sophisticated entity that, when in good health and functioning well, it will exhibit fractal patterns and chaotic or complex patterns of structrure and change. Some of this complexity diminishes during the aging process, presumably indicating that the organism is losing the capacity to control the multi-dimensional, conditional, and dynamic processes underlying the complexity. Loss of complexity is not necessarily based on a network of molecular interactions. In fact, the primary examples of loss of complexity are traits such as heart rate variability and branching structure of vessels (Lipsitz and Goldberger 1992), traits that are not well explained through a network understanding of organisms. Loss of complexity may at least partly explain or characterize aging, and it may influence or be influenced by aspects of complex system dynamics.

## Complex systems dynamics and physiological organization

Most research on physiological regulation today proceeds molecule by molecule. The basis of this reductionist approach is the idea that if we can understand the role of each molecule in regulating other molecules and being regulated by them, we will be able to understand how physiological systems or entire organisms function. This approach is not without merit—many successful pharmaceutical products have been developed by understanding molecular interactions and developing compounds to intervene in these interactions. However, most potential pharmaceutical products never pan out, and many that do are found to have unexpected side-effects, short or long-term (Ahn et al. 2006; Jüni et al. 2004). Though it would be a mistake to put too much emphasis on the reductionist-holist distinction (Kirkwood 2011), this reflects certain limits of a purely reductionist approach.

To understand why this is, we need a coherent model of how physiological systems are structured and how they evolve. A starting point is the *raison d’être* of these systems: they exist to help organisms maintain homeostasis, and to adjust this homeostasis as necessary in response to changing environmental conditions or internal physiological conditions (Cohen et al. 2012; Kitano 2007; Kriete 2013). The term “homeostasis” is imperfect in this context, given that organisms are dynamic entities in constant flux. Here, I do use the term homeostasis, but with a broad conception that what is static is not the state of the molecules, but that the organism is maintained in or adjusted to whatever physiological state may best serve its interests at the moment. This is consistent with its original definition (Cannon 1932), despite later criticism. Organisms achieve homeostasis through robustness to perturbations (Kitano 2007). For example, our diet changes slightly from day to day; it would not do for small changes in intake of vitamins, phytoestrogens, etc. to cause major shifts in physiological state. At the same time, physiological state does need to change in coherent ways—for example, to enter a breeding state, to digest a meal, to mount a stress response, or with circadian rhythms. Organisms thus need physiological systems that are largely robust to minor perturbations, and that can make coherent shifts in physiological state as necessary. Moreover, if a problem arises in the regulation, there is no external force that can intervene to restore homeostasis. Accordingly, both robustness and desired shifts in physiological state must result directly from the organization of molecules in a regulatory network.

**Fig. 2:**
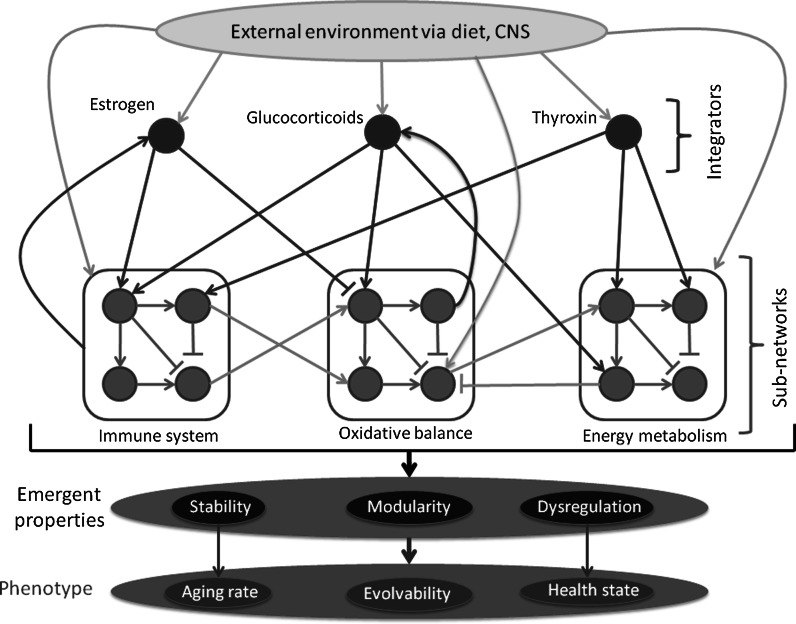
A simplified, partial schematic of a physiological regulatory network (PRN). *Red arrows* indicate top-down control, such as steroid hormone modulation of immune function. *Purple arrows* indicate feedback effects, such as antioxidant effects on glucocorticoids. *Light-blue arrows* indicate direct interactions among subnetworks, such as immune regulation by dietary antioxidants. *Green arrows* indicate direct effects of the environment on subnetworks, such as content of antioxidants in the diet. *Yellow arrows* indicate environmental regulation of integrators, usually via the central nervous system (CNS). System-level properties of the PRN exist at different levels, including state within individuals (e.g. dysregulation) and species-level structure (modularity). Likewise, phenotype can include individual- or species-level traits (e.g. health and evolvability, respectively). Modularity is determined by the proportion of potential *light-blue arrows* present; interconnectedness by the total number of *arrows* relative to molecules; and robustness by the density of *purple arrows* resulting in negative feedback effects. The particular structure of connections, as well as their strengths and interactions, will determine how the PRN responds at an individual level and evolves at the species level in response to a changing environment. Adapted from Cohen et al. (2012)

At the level of the organism, I call these “physiological regulatory networks” (PRNs, Fig. 2) (Cohen et al. 2012). Within an organism, all biologically active molecules can be considered part of a single large PRN. Figure 2 is a simple caricature – there are hundreds if not thousands of sub-networks, and many molecules have yet to be discovered. The key point of the figure is the structure of regulation: it is not simply hierarchical (from the top down), but also bottom-up and with substantial direct cross-talk among systems. For example, vitamin E has important roles in both maintaining oxidative balance and in the immune system (Chew 1995). The bottom-up effects and cross-talk create feedback loops, a key structural feature of networks that helps them maintain homeostasis (e.g. through negative feedback) or shift physiological states (e.g. through positive feedback) (Cinquin and Demongeot 2002; Wiener 1961). Another key structural feature of PRNs is redundancy (Kitano 2002). Redundancy helps ensure that problems in one small part of the PRN can be contained, and thus that the PRN is not overly sensitive to minor perturbations.

These structural features mean that PRNs can be formally considered complex dynamic systems (Holland 1992; Kier and Witten 2005), much like weather systems and ecological networks (e.g. Dunne et al. 2002). However, PRNs have one key difference from many other complex systems: they have been shaped by natural selection for a specific purpose, i.e. to maximize organismal fitness (Cohen et al. 2012). Even undirected networks such as weather systems and ecological networks can demonstrate coherent structure; this is a result of self-organizing properties that can emerge in complex systems (Kauffman 1993). But in PRNs and other biochemical networks this organization should be particularly clear and should relate directly to traits that influence fitness.

Indeed, what we know about PRN structure strongly supports this model. While some molecules may play key roles coordinating function across systems (integrators, Fig. 2) (Martin et al. 2011), PRN function is relatively robust to perturbations in levels of these integrators. For example, polybrominated diphenyl ethers (PBDEs) are endocrine disruptors that have been shown in animal models to be able to disrupt growth processes by mimicking thyroid hormones (Suvorov et al. 2008; Zhou et al. 2001). Nonetheless, in human epidemiological data there is no evidence for an effect of PBDEs on growth rates. Increased PBDE levels result in decreased thyroid levels (Abdelouahab et al. 2013), but no change in birth weight (Y. Serme and A.A. Cohen, unpubished data). This suggests that thyroid hormone production decreases as a response to PBDEs, and that up to a certain point PBDEs simply replace thyroid hormone. Only when levels are particularly high or other factors contribute does major physiological disruption occur. This is a good example of how control of PRNs is diffuse: only rarely does a single molecule exert exhaustive control over a process, and often redundancy and feedback combine to assure a functional stability. Additional support for this is seen in the failure of many gene knockouts to produce major changes in phenotype (Barbaric et al. 2007).

The model of physiological organization outlined here is not revolutionary—in fact, most of its elements can be found in introductory biology courses, to say nothing of systems biology textbooks and reviews (Kitano 2002; Klipp et al. 2005). Nonetheless, its consequences are important. It suggests that the best way to understand physiological system state is neither by using single molecules to describe system state, nor by creating an exhaustive map of all molecules and their relationships. Rather, key aspects of system state might be measured with small numbers of molecules, and the precise choice of molecules might not be that important, because system state is a diffuse property of the network as a whole. Any single molecule might measure this state with a large amount of error, but as the number of molecules increases so does the signal, with diminishing returns for each additional molecule. This is encouraging: it implies that with relatively simple multivariate statistics applied to small numbers of markers (<50) we may be able to decode how PRNs are structured and to measure the state of an individual. This model also suggests that complex systems dynamics may be key to understanding aging, i.e., that aging may be to some degree an emergent property of PRN function. We wanted to understand to what extent these kinds of dynamics are important in physiology generally, and in aging specifically.

## Emergent physiological processes

### Overview

The general framework just described suggests that physiology may be organized in part around what I call EPPs. An EPP is a process controlling a key aspect of regulation through dynamic interactions among large numbers of molecules in a way that cannot be easily understood by mapping the direct regulatory relationships among the molecules. Emergence in physiological systems is generally discussed in terms of emergent properties: robustness, modularity, etc. However, there is good theoretical reason to suspect that emergence can occur for processes as well as for properties. Unlike emergent properties, which can often be measured in a standardized way across different types of networks, emergent processes are specific to the functional objectives of a given network. For example, both ecological networks and physiological networks might be highly modular (a property), but it makes no sense to discuss regulation of systemic inflammation (a process) in an ecological network.

The concept of EPPs has not, to my knowledge, been previously described in a way specific to physiological regulation, though in complex systems theory more generally the existence of such processes was predicted over 20 years ago by Kauffman (1993). Kauffman used simulations to show that complex dynamic systems can have clear attractor states related to attractor basins: within a multivariate state space, there exist a number of distinct regions within which regulatory networks cause a dynamic process that leads the system state to converge on a single point, such that even though the number of starting points may be enormous, there are a limited number of convergence points to which the system tends. This is the equivalent of the tendency of water to flow to the lowest point in a drainage basin: the number of local valley bottoms is limited, even on a large landscape. (The analogy breaks down due the presence of water bodies such as rivers and lakes that stop the descent.) The idea of attractor states fits well with the general model of physiological regulation above because these states are likely to correspond to key situations organisms encounter, such as shifts between breeding and non-breeding states.

Of course not all states that organisms need to arrive at are discrete, and it is not hard to develop a conceptual generalization of the attractor state model to incorporate continuous variation in an organism’s physiological state. Aging is a good example of this. Many aging-related changes in physiology are likely adaptations to other aging-related changes, adaptations that minimize the impact of aging on the organism. Such adaptation likely needs to be continuous rather than discrete, and the analogy would thus be to attractor “trenches,” where there is not a single point to which physiology converges, but rather a series of points along a continuous axis. The system will be able to converge to different points along this axis in response to some additional control factor. Note that this axis or trench could align with many physiological parameters to reflect the continuous adjustment of many different aspects of physiology in a coordinated fashion.

As mentioned above, if control of which state an organism is in depends too heavily on any single parameter, the organism is at substantial risk of a regulatory error should that parameter become abnormal for any reason. Accordingly, we should expect that control of shifts among attractor basins occurs in a more sophisticated way involving feedback loops and redundancy across many parameters. This would reflect selection for PRN structures that increase robustness to common problems while decreasing robustness to rare problems (Kriete 2013).

Putting all this together, we should expect that shifts among attractor states and/or along attractor trenches would often be regulated by complex feedback mechanisms occurring among many molecules, and that mapping the regulatory pathways of the molecules would not necessarily give us clear insight into what the attractor states/trenches are nor into how those shifts result from the regulatory pathways. Obviously, we neither expect nor observe that such complex mechanisms are the only mechanisms of physiological control. For example, insulin signaling controls glucose metabolism in a rather straightforward way, and complex systems theory is not necessary to explain the functional significance of this pathway. Nonetheless, almost all research into physiological regulation to date has been conducted as if all key processes can be detected in the same way as insulin signaling, and there is good reason to believe that EPPs might exist and might control numerous important processes for maintaining and adjusting homeostasis.

### Evidence

Some of our recent findings provide the first clear empirical support for the existence of EPPs. In full disclosure, we did not predict the existence of EPPs and then confirm this empirically, but rather obtained unexpected results and developed the concept of EPPs as the most coherent explanation for these findings. The principal result was the detection of unexpected yet highly stable associations among a number of biomarkers (Cohen et al. 2015b). We had used the statistical method principal components analysis (see “Principal Components Analysis (PCA)”) to try to understand links among 43 common clinical biomarkers during aging from the Women’s Health and Aging Study (WHAS), expecting to be able to simplify our dataset into summary measures of known systems. Instead, the most important axis (i.e., PCA 1) cut across traditional classifications of biomarkers, associating particularly strongly with those relating to anemia, protein transport, inflammation, and calcium. Thinking we had perhaps made an error or that our result was due to random processes in the data, we replicated it in two additional datasets, InCHIANTI and the Baltimore Longitudinal Study on Aging (BLSA), as well as in multiple demographic subsets of each dataset (male/female, black/white, younger/older, etc.). In each case we were able to faithfully replicate the axis, with versions calculated from different datasets and subsets generally producing scores correlated at *r* > 0.9, often >0.95.

### Principal Components Analysis (PCA)

Principal components analysis (PCA) is a data reduction method that can be used to condense a large number of redundant variables into a smaller number of independent variables. While PCA is a standard method, it has a rarely appreciated potential to identify underlying processes structuring the data (e.g. Cohen et al. 2010). For example, if we applied PCA to a data set of thousands of individuals for whom we measured 100 morphologic traits (height, weight, arm length, leg length, waist circumference, nose width, etc.), we would expect to be able to greatly simplify the dataset. Arm length, leg length, and height are tightly correlated and thus largely redundant. We thus might expect the first composite variable (“axis”) generated by the analysis to be an overall measure of size, positively associated with all our measures. The second axis might be a measure of skinniness/obesity, with measures like height, arm length and leg length juxtaposed against measures like waist circumference, arm circumference, weight, etc. We would likely have several other important axes as well. These axes would provide a useful summary of the data: 5–10 variables is easier to manage than 100. But critically they would also provide insight into the biological processes determining morphology. If a certain gene controls aspects of development that cause nose width and finger circumference to covary, this would show up in our axes. If fat composition in the diet systematically affects where fat is deposited, this will also show up in our axes. Careful interpretation of PCA axes can thus yield important substantive insight into underlying processes structuring the data.

It was clear that the physiological axis we had detected in WHAS, InCHIANTI, and BLSA represented some underlying process structuring the correlations among the biomarkers in a consistent way across populations and sub-populations, but it was not yet clear if this process was biologically interesting. We replicated the analyses after controlling each biomarker for age and obtained the same signal again, confirming that we were not measuring some proxy for age. We tested for an association with hepcidin, a recently discovered hormone thought to be important in regulation of some of the systems associated with our process (Nemeth et al. 2004), but the correlation was weaker than for most of the individual biomarkers used. We tested for the stability of the axis across datasets compared to the stability of the individual biomarkers, both in terms of correlations with age and correlations with each other, and showed that the axis is more stable than any of the individual biomarkers. At this point, we concluded that the most likely explanation for the process we detected is that it represents an axis of physiological regulation integrating multiple systems, and that appears to function outside the direct regulatory control of any single molecule or pathway. We noted that it appears to increase exponentially with age, and that it predicts mortality and clinical frailty (but not chronic diseases) after controlling for age. In other words, it appears to be an EPP that is implicated in aging but not chronic diseases.

While the process we detected, which we call “integrated albunemia” (PCA1, no relation to albuminenia), appears to be a clear example of an EPP, it is not the only likely example. The second axis from the same analysis appears to represent metabolic syndrome based on strong associations with lipids, glucose, and inflammation (Cohen et al. 2015b). Metabolic syndrome (Grundy et al. 2004) fits the definition of an EPP presented above. Likewise, inflamm-aging (a suite of changes in inflammatory regulation with age, Franceschi et al. 2000) appears to represent another EPP. In another recent study, we showed that inflamm-aging is characterized not simply by up-regulation of pro-inflammatory markers, but by simultaneous up-regulation of both pro- and anti-inflammatory markers, suggesting that it represents a coherent shift in system state rather than a clear outcome of a simple regulatory pathway (Morrisette-Thomas et al. 2014).

All three of these examples of potential EPPs are still tentative, in that we cannot definitively exclude the possibility of simple molecular control mechanisms, nor of other potential explanations. Nonetheless, for the reasons listed above, there is good reason to suspect that EPPs might exist, and these examples appear to exhibit the predicted properties. Interestingly, all three represent either pathological changes in system state or adaptations to pathological changes (it is not easy to distinguish which). Nonetheless, there is no reason to suspect that EPPs would be limited to progression of aging-related pathologies; indeed, we would predict that if EPPs are a common feature of physiological organization, they should also exist to help organisms transition along “attractor trenches” even when no point along the trench represents a more pathological state than any other.

## Physiological dysregulation

### Overview

The second major branch of our research has focused on quantifying physiological dysregulation. In some sense, physiological dysregulation during aging is trivial: clearly, many aspects of physiology and regulation function less well during the aging process. However, we use the term in a more restricted and less trivial sense, referring specifically to a gradual and generally irreversible loss of regulatory control originating from structural instabilities in regulatory networks. All complex systems have some tolerance limits to changing conditions. For example, there exist temperatures, blood glucose levels, etc. that are simply and immediately fatal for an organism. As mentioned above, highly optimized complex systems have generally evolved to tolerate as wide a range of common conditions as possible, while remaining frail/susceptible when faced with more abnormal conditions (Kriete 2013). The question is, what happens when a system/organism is pulled slightly outside its optimal tolerance range? Does it either die immediately, or survive as if nothing happened? Or does it survive, but with its overall system state slightly modified and unable to fully return to complete homeostasis? If this latter possibility exists (and it is by no means clear it does), that would be what I call physiological dysregulation. Note that this model does not necessarily imply that aging/dysregulation could be avoided simply by maintaining perfect conditions, because organisms may not have perfect tolerance for the varying internal conditions that they will inevitably undergo during their life course.

Unlike with EPPs, we hypothesized a priori that physiological dysregulation is a major driver of the aging process, perhaps even largely sufficient in some cases to explain most of aging as we know it. We thus set out to find ways to detect a signal of physiological dysregulation. We started with the Anna Karenina principle: “All happy families are happy in the same way, but each unhappy family is unhappy in its own way.” Taking the analogy to physiology, all well-regulated systems are relatively similar, but there are a multitude of ways in which things can go wrong. This analogy was first proposed to me by Arun Karlamangla. Using this principle, we hypothesized that individuals with a normal biomarker profile should generally be healthier and younger than those with an abnormal profile.

### Evidence

**Fig. 3:**
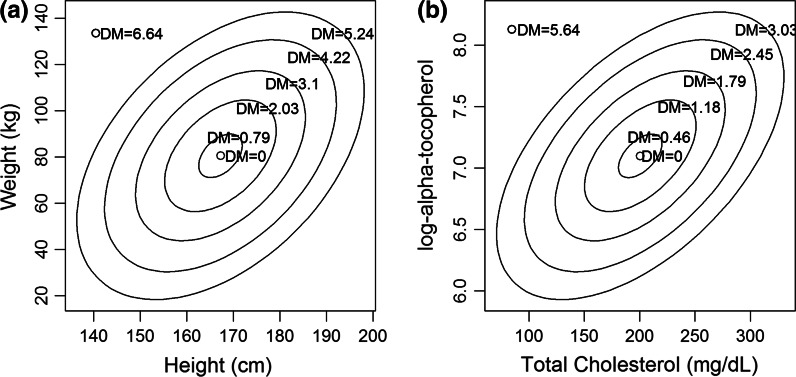
A general, 2-dimensional example of Mahalanobis distance (D_M_) based 30,000 + adults in the NHANES dataset. **a** gives the relationship between height and weight (an intuitive example), and **b** between total cholesterol and vitamin E (two biomarkers in our data sets). The correlations between these variables are *r* = 0.45 and 0.54, respectively. The concentric ellipses represent, from inside to outside, ellipses that should contain 0, 10, 50, 80, 95, and 99 % of the observations, based on the combination of the correlation, means, and standard deviations. D_M_ here reflects how rare any height-weight or cholesterol-vitamin E combination is, and thus has an equal value for all points on the same ellipse, as indicated in *red*. Because D_M_ incorporates the correlation into the calculation, it reflects the fact that certain *combinations* may be more unusual than expected based solely on how rare the values are separately. For example in **a**, the point in the upper left (height = 140 cm, weight = 133 kg) has a D_M_ of 6.64, substantially higher than D_M_ = 5.24 for the point on the 99 % ellipse in the upper right (height = 194 cm, weight = 133 kg) *despite the fact that heights of 140 and 194* *cm are equally rare in the population* (99.6th percentile). Accordingly, D_M_ correctly reflects the fact that it is much rarer to be short and heavy than tall and heavy. In practice, D_M_ applies this principle to large numbers of variables simultaneously, though visualization is hard beyond two dimensions

In order to measure how normal a profile is, we applied a measure of statistical distance. Statistical distance is a way to quantify how different a vector of values (e.g. a biomarker profile) is from some standard profile. Specifically, we used the Mahalanobis distance (D_M_) as a measure of how normal or abnormal an individual’s overall biomarker profile is (Cohen et al. 2013; Mahalanobis 1936). Because biomarkers are correlated, it is not possible to evaluate how unusual a profile is based solely on how unusual each biomarker is. For example, it is about equally rare for adults in the US to be 194 cm tall or 140 cm tall, but the combination of 140 cm with 133 kg is much rarer than the combination of 194 cm with 133 kg (Fig. 3a). Similar principles apply to biomarkers (Fig. 3b). Standard clinical and research approaches to biomarkers consider them one at a time, but D_M_ provides a simple way to adjust for their joint probability distribution. We hypothesized that, under the PRN model above, having an abnormal or unusual biomarker profile as measured by D_M_ would be a sign of physiological dysregulation (i.e., deviation from homeostasis), and that physiological dysregulation in turn was important in aging. We made a number of specific predictions to test this hypothesis:

1. D_M_ should increase with age.
2. High D_M_ should be associated with higher mortality and adverse health outcomes, controlling for age.
3. The signal of D_M_ should increase as more markers are used to calculate it, but with diminishing returns for each additional marker.
4. The precise choice of markers should not matter too much, as long as they are broadly representative of the system or organism in question.
5. The signal of D_M_ should increase if the “normal” profile is calculated based on a relatively young, healthy sub-population.
6. These results should be broadly replicable and stable across populations and species.

**Fig. 4:**
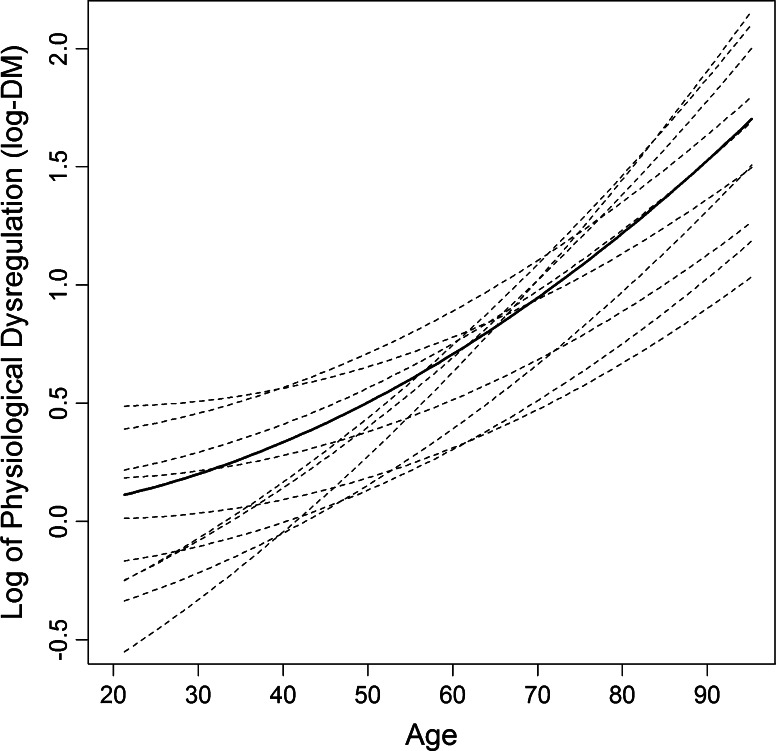
Estimated trajectories of log-D_M_ with age at the population (*solid black line*) and individual (*dotted lines*) levels for the InCHIANTI cohort based on quadratic Bayesian multi-level models. These models estimate an overall (population) quadratic function for change in D_M_ with age, as well as individual deviations from this function. Each individual’s trajectory is estimated with substantial error, but overall estimates of the heterogeneity of trajectories are robust with the sample sizes available. Individual trajectories are shown for ten individuals selected randomly in the dataset as an example. Inference is based on statistical distance of 43 common clinical biomarkers (albumin, glucose, cholesterol, etc.) measured in 1022 individuals aged 21–96, with up to four visits per individual. While individual heterogeneity in level and rate of change in D_M_ is significant, the general trend toward increasing and accelerating D_M_ with age is also clear. More complete analyses and details are available in Milot et al. (2014b)

Indeed, we can now confirm all of these quite convincingly using data on 44 common clinical biomarkers from the same aging cohort studies mentioned above in the PCA analyses (Cohen et al. 2013, 2014, 2015a; Milot et al. 2014a, b). D_M_ increases with age, probably exponentially, and the curves are consistent across WHAS, BLSA, and InCHIANTI (Fig. 4; Milot et al. 2014b). A very different group of markers gives a very similar pattern in a fourth cohort study, NuAge, based in Quebec (unpublished data). D_M_ predicts mortality and clinical frailty after control for age. It is also associated with a number of chronic disease measures—very consistently with total number of comorbidities and heart disease, somewhat less consistently with diabetes, and only rarely for cancer (Cohen et al. 2014, 2015a; Milot et al. 2014b).

Many of our analyses have been replicated across a very large number of biomarker combinations. Our initial analyses used a group of 14 markers identified through a statistical selection procedure, and were replicated on every combination of these 14, i.e. 16 383 combinations (Cohen et al. 2013), (2014). We then used the full set of 44, testing 5000 random combinations for each possible number between 1 and 44, or all combinations when less than 5000 existed (Cohen et al. 2015a). We consistently found that including more markers produced a stronger signal, but with diminishing returns, as predicted. We showed that two mutually exclusive groups of biomarkers still tended to produce D_Ms_ that correlated relatively well (*r* = 0.4–0.5 when n = 20 per group). There was moderate heterogeneity in results across combinations: for example some predicted one outcome more strongly, others another. Nonetheless, the general tendencies were consistent. Few enough combinations were associated with cancer that this could be due solely to sampling processes and false positives; cardiovascular disease was almost always positively associated with D_M_, though sometimes not significantly depending on the combination.

In addition to validating these results in four human datasets, we applied the same technique to a dataset on 11 biomarkers measured monthly in 32 captive red knots (*Calidris canutus*), a shorebird (Milot et al. 2014a). D_M_ was clearly predictive of the two health/performance measures available, a foot inflammation score (positive association) and maximum aerobic capacity (negative association). The 11 biomarkers had already been measured for other reasons, showing that the principal can be applied to biomarkers chosen more-or-less randomly.

Lastly, we have recently shown that D_M_ can be calculated not just globally, at the organism level, but also meaningfully for distinct physiological systems (Li et al. in press). D_M_ calculated based on biomarkers for lipid, electrolyte, oxygen transport, vitamin, liver function, and white blood cell types showed the same traits as global D_M_, but D_M_ in each system was only weakly correlated with D_M_ in the others after control for age. This suggests that dysregulation may proceed largely independently within each system, but with the potential for feedback effects such that a global level can also be meaningfully measured. This result is crucial for our ability to take inference about dysregulation to a finer biological scale than the whole organism, and suggests a long-term direction for understanding how lower-order processes produce higher-order ones during aging.

It is surprising that our results so consistently and strongly confirm the utility of D_M_ as a measure of dysregulation, given how crude the measure is. It requires us to assume (a) that the ideal biomarker profile is the average profile; (b) that this ideal profile is identical for all individuals at all ages, sexes, and physiological states, and (c) that the distribution of profiles in biomarker space is multivariate normal. All three of these assumptions are clearly false, and may sometimes not even be approximately true. We are working on better ways to estimate of the centroid (i.e., optimal profile) and to relax the supposition of multivariate normality (e.g. Ekström 2011; Liu et al. 1999). The fact that D_M_ works well despite the crudeness of these assumptions suggests that the true signal (i.e., the signal we would detect with an optimal method) is very strong and biologically important.

Taken together, these results show that D_M_ does measure physiological dysregulation as an emergent property of system state, and that physiological dysregulation is an important part of the aging process. They do not, however, show whether dysregulation is a primary cause of aging or a result of other, upstream processes. The robustness to choice of biomarkers is strong enough to confirm the general physiological model proposed, but weak enough to imply that greater detail of specific processes will be important to incorporate going forward.

## Discussion

 Overall, our research to date provides convincing but not yet ironclad evidence for a role of complex systems dynamics in aging. Our findings on integrated albunemia, metabolic syndrome, and inflamm-aging are highly consistent with EPPs, and thus suggest a model of physiology in which function is not necessarily determined through distinct, clearly decipherable molecular pathways, but also through complex interactions among large numbers of molecules, at least in some cases. For all three potential EPPs discussed here higher scores indicate worse health. This may indicate that they are pathological processes, or that they are adaptive responses to other changes in system state with age, or a combination of the two. However, there may also be other EPPs that are not involved in aging at all, and that help organisms control transitions among key physiological states.

Likewise, physiological dysregulation appears to be a separate type of complex systems dynamic involved in aging. When we began our study, it was not clear whether more-or-less random combinations of 10–15 common biomarkers could provide a coherent signal of overall system state. Many of these markers are not themselves correlated with age in any consistent way (Cohen et al. 2015b). Our findings thus provide clear support for a specific definition of physiological dysregulation: as a gradual inability of physiological systems to return to a baseline (e.g. homeostatic) state due to complex interactions at the system-level. Obviously, there is no single optimal physiological state to which all organisms attempt to return (Kitano 2007); the optimal state must shift with conditions, and substantial work remains to outline how dysregulation measures interact with an organism’s changing physiological needs. However, this limitation makes it all the more surprising that we do consistently find that a simple measure of how aberrant a biomarker profile is consistently predicts everything we would expect from a measure of biological age (Klemera and Doubal 2006; Levine 2013). Despite this, I would take a cautious approach relating D_M_ to biological age. The semi-independent dysregulation in different systems we find means that there is no single, universal definition of biological age. Biological aging is likely multi-dimensional, and this is reflected in the imperfect correlations among D_M_ generated from different biomarker combinations.

It would appear that our analyses present the first clear demonstration of generalized physiological dysregulation as a system-level property during aging. This finding is unsurprising, given a number of excellent previous studies. For example, Fried et al. (2009) showed non-linear changes in biomarkers across systems during aging. Govindaraju et al. (2014) showed complex interactions among markers of cardiac function during aging. Arbeev et al. (2011) showed that individual biomarkers exhibit a loss of homeostatic dynamics during aging. Yashin et al. (2010) showed that dynamics of biomarkers during aging may be at least as important as static levels. Dan Belsky (pers. comm.) showed that changes in biomarker profiles characteristic of aging start early, at least by the 30 s in humans. Southworth et al. (2009) showed that gene expression profiles in mice become increasingly uncorrelated with age.

Physiological dysregulation as we detect it is consistent with many of the more clinical conceptualizations related to homeostasis (Ferrucci 2005; Fried et al. 2005; Seplaki et al. 2005). In particular, some of the mathematical properties of the robustness to biomarker choice resemble those of the frailty index (Howlett et al. 2014; Searle et al. 2008) and suggest that D_M_ may be detecting the frailty process well before it reaches clinical manifestations. This idea is somewhat at odds with the idea of integrated albunemia as the basis of frailty, and considerable work remains to identify which if either of these processes is more important in frailty’s etiology, or even whether frailty represents a coherent biological process.

Our work has various links to the other approaches to complexity in aging outlined above. Complex systems dynamics such as we detect do not contradict the possibility for more additive, multi-factorial processes (Kirkwood 2005; Weinert and Timiras 2003). The underlying model of physiology is quite similar to the model of cellular regulation supposed by Kriete (2013) in describing the role of robustness in aging. His approach is complementary to our approach in that it uses the principle of robustness to arrive at general insights into trade-offs and the evolution of aging; we attempt to quantify functional outputs of complex system dynamics at the individual level, with potential implications at evolutionary scales. The relationship between loss of complexity (Lipsitz 2004; Lipsitz and Goldberger 1992) and our approach to network state and dysregulation is hard to discern intuitively, and empirical studies will likely be needed to establish any links. Our results would appear to suggest that there may be both gains and losses of complexity with aging: the increases in D_M_ we observe with age demonstrate greater variation in physiological state with age. The question then becomes, is variation in these traits equivalent to complexity? There is substantial potential for research to bridge these approaches, such as studies examining how short-term temporal variation in D_M_ changes with age.

Most of our research to date has been at the organismal level, as has the research on physiological dysregulation more broadly and on loss of complexity. In contrast, much of the work on multi-factorial aging and on robustness focuses on cellular aging. One of the major challenges going forward is to understand how these levels interact, and whether one has primacy over the other in the aging process. One hypothesis would be that cellular aging causes organism-level physiological dysregulation, but that variation in aging across species depends on the species-level robustness of organism-level PRNs to withstand this dysregulation. The statistical approaches we have developed to measure complex system dynamics could also be applied to cellular networks, and it will be important to assess whether cellular dysregulation is linked to cellular senescence in the same way that organism-level dysregulation appears related to organismal senescence.

Overall, it is becoming increasingly clear that complexity generally, and complex network dynamics specifically, play important roles in the aging process. It remains to be seen exactly what these roles are, and to what extent other processes are important as well. I suspect but cannot yet prove that dysregulation is itself a major driver of the aging process. If this is correct, there are substantial impacts for understanding both the mechanisms and evolution of aging. Aging rates across species would be determined largely by their ability to resist dysregulation. There are also impacts for medical research on aging: if dysregulation of complex networks is a crucial aspect of aging and is universal in mammals, there is little hope that rejuvenation therapies will be able to do much more than serve as a speed bump during the aging process. Conversely, understanding how lifestyle and genetic background affect dysregulation rates may offer substantial hope for improving health span.

I have little doubt that there are other fruitful approaches to the complex systems dynamics of aging waiting to be explored. Research to date has barely scratched the surface, and a combination of recent findings and basic principles of biological organization suggest that these dynamics play an important role in structuring the aging process, both on a mechanistic and an evolutionary scale. If there is anything we have learned after decades of research on aging mechanisms, it is that there is unlikely to be any silver-bullet explanation, even if that explanation is relatively broad, such as complex systems dynamics. Nonetheless, this field remains one of the most promising and least explored aspects of aging.
